# Pollinator population size and pollination ecosystem service responses to enhancing floral and nesting resources

**DOI:** 10.1002/ece3.2765

**Published:** 2017-02-19

**Authors:** Johanna Häussler, Ullrika Sahlin, Charlotte Baey, Henrik G. Smith, Yann Clough

**Affiliations:** ^1^ Centre for Environmental and Climate ResearchLund UniversityLundSweden; ^2^ Department of Biology, BiodiversityLund UniversityLundSweden; ^3^ Present address: Johanna HäusslerGerman Centre for Integrative Biodiversity Research (iDiv) Halle‐Jena‐LeipzigLeipzigGermany; ^4^ Present address: Institute of EcologyFriedrich Schiller University JenaJenaGermany; ^5^ Present address: Charlotte BaeyCentraleSupélecMICS LaboratoryChâtenay‐MalabryFrance

**Keywords:** bee foraging, bumble bees, crop pollination, ecosystem service mapping, landscape ecology, natural capital, pollination model, population dynamics, wildflower habitat

## Abstract

Modeling pollination ecosystem services requires a spatially explicit, process‐based approach because they depend on both the behavioral responses of pollinators to the amount and spatial arrangement of habitat and on the within‐ and between‐season dynamics of pollinator populations in response to land use. We describe a novel pollinator model predicting flower visitation rates by wild central‐place foragers (e.g., nesting bees) in spatially explicit landscapes. The model goes beyond existing approaches by: (1) integrating preferential use of more rewarding floral and nesting resources; (2) considering population growth over time; (3) allowing different dispersal distances for workers and reproductives; (4) providing visitation rates for use in crop pollination models. We use the model to estimate the effect of establishing grassy field margins offering nesting resources and a low quantity of flower resources, and/or late‐flowering flower strips offering no nesting resources but abundant flowers, on bumble bee populations and visitation rates to flowers in landscapes that differ in amounts of linear seminatural habitats and early mass‐flowering crops. Flower strips were three times more effective in increasing pollinator populations and visitation rates than field margins, and this effect increased over time. Late‐blooming flower strips increased early‐season visitation rates, but decreased visitation rates in other late‐season flowers. Increases in population size over time in response to flower strips and amounts of linear seminatural habitats reduced this apparent competition for pollinators. Our spatially explicit, process‐based model generates emergent patterns reflecting empirical observations, such that adding flower resources may have contrasting short‐ and long‐term effects due to apparent competition for pollinators and pollinator population size increase. It allows exploring these effects and comparing effect sizes in ways not possible with other existing models. Future applications include species comparisons, analysis of the sensitivity of predictions to life‐history traits, as well as large‐scale management intervention and policy assessment.

## 1 Introduction

Predicting the impact of land‐use change using ecological production functions that link ecological characteristics in a given area to the production of one or multiple ecosystem services is important to inform policymakers and practitioners (Larigauderie & Mooney, 2010; Wong, Jiang, Kinzig, Lee, & Ouyang, 2015). While substantial efforts are made to map ecosystem services (Maes et al., 2012), significant challenges such as identifying data gaps and developing models and ecological production functions remain. This applies in particular to ecosystem services provided by mobile beneficial organisms such as pollinating insects where the consequences of management play out at landscapes scales (Kremen et al., 2007).

Insect pollination is vital to the functioning of both natural and human‐modified ecosystems. As reviewed by Klein et al. (2007), the production of approximately 70% of the major global crops depends to some extent on animal pollination. Besides the European honey bee (*Apis mellifera*), wild bees (Hymenoptera: Apoidea) are important pollinators for many of these crops (Garibaldi et al., 2013). Wild bees however have suffered from past increases in field sizes, specialization toward arable farming, loss of flower‐rich seminatural habitats, and increased use of agrochemicals, with negative effects on wild bee diversity and abundances, shifts in community composition, and reduced pollination ecosystem services to crops (Bommarco, Lundin, Smith, & Rundlöf, 2012; Potts et al., 2010; Vanbergen & Insect Pollinators Initiative, 2013).

To counteract the decline of wild pollinators and bolster pollination ecosystem services, enhancing floral resources and increasing suitable nesting habitat have been recommended (Blake, Westbury, Woodcock, Sutton, & Potts, 2011; Holzschuh, Dudenhöffer, & Tscharntke, 2012). This may include setting aside land from cultivation, preserving grasslands and field edges, and sowing wildflower patches or strips (Jönsson et al., 2015; Scheper et al., 2013). The link to ecosystem services and natural capital, however, is often challenging to assess due to the complex spatiotemporal processes that govern pollinator foraging and population‐level responses. For example, the effect of providing additional food resources might be complex, as they may benefit wild bee populations on the long term, but at the same time draw bees away from crops on the short term (Hegland, 2014; Olsson, Bolin, Smith, & Lonsdorf, 2015). Further, grasping the scale at which pollinator populations respond to increases in resources is difficult because foraging of workers and dispersal of reproductives occur at different spatial scales (Lepais et al., 2010). In other instances, mass‐flowering crops such as oilseed rape (OSR) may increase forage resources for pollinators, but high fluctuations in the cover of annual crops may create spatial source–sink dynamics and prevent build‐up of pollinator populations (Riedinger, Renner, Rundlöf, Steffan‐Dewenter, & Holzschuh, 2014; Rundlöf, Persson, Smith, & Bommarco, 2014; Westphal, Steffan‐Dewenter, & Tscharntke, 2003).

Spatially explicit, process‐based ecological production functions based on fundamental knowledge of pollinator behavior and population dynamics have the potential to take these effects into account (Olsson et al., 2015; Rands, 2014), and enhance transferability compared to statistical approaches. This would allow for simulation‐based evaluation of management interventions, useful as a complement to field studies, because replicated long‐term experimental landscape‐scale management interventions are generally difficult to conduct. The models currently in use to map pollination ecosystem services (Lonsdorf et al., 2009; Zulian, Maes, & Paracchini, 2013) estimate pollination ecosystem services using distance from likely nesting sites and therefore ignore resource‐dependent optimal foraging by pollinators and its consequences on population dynamics such as those observed by Riedinger et al. (2015), and provide only relative measures of pollinator visitation to crops. More realistic predictions of visitation rates may be achieved by basing the model on foraging theory (such as Olsson et al., 2015) and by including population dynamics (Crone & Williams, 2016) within and between years.

**Figure 1:**
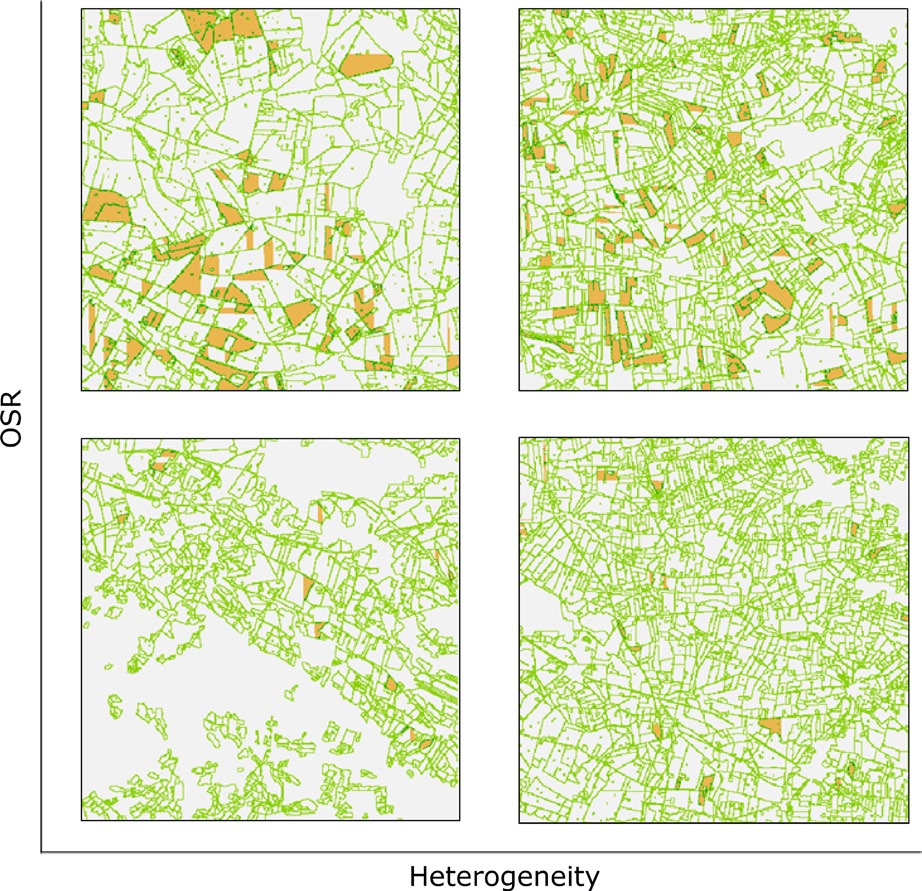
Four exemplary landscapes used in this study with a varying amount of oilseed rape fields (orange) and different structural heterogeneity of fields (green)

Here, we develop a pollinator model that (1) integrates preferential use of more rewarding floral and nesting resources; (2) considers population growth within and between years; (3) allows for different movement distances for foraging and queen dispersal (Lepais et al., 2010); and (4) produces spatially explicit flower visitation rates. We demonstrate the model by applying it on common early‐active bumble bees, using parameters based on published data and expert opinion, and GIS rasters of real agricultural landscapes. We use the model to quantify the long‐term impacts of enhancing nesting resources by widening grassy field margins and/or enhancing floral resources by sowing late‐flowering flower strips. In addition, we assess the extent to which effects depend on landscape‐scale cover of mass‐flowering crops and structural heterogeneity (OSR and HET; Figure 1), and how they change over time.

## 2 Materials and Methods

### 2.1 Simulation environment and land‐use information

The impact evaluation was set up as a simulation experiment running the model described below for 20 10 km^2^ agricultural landscapes from Scania, southern Sweden, selected to obtain uncorrelated gradients of landscape heterogeneity and oilseed rape cover. Using land‐use data combined with yearly field‐level crop‐cover data from 2007 to 2013, we generated two rasters with 25 × 25 m resolution for each landscape and year, one containing land‐use and crop‐cover codes, the other containing the length of agricultural field block edges per cell. A field block is a field or a group of fields that is surrounded by uncultivated field borders. Nesting and floral resource raster layers were derived from the land use/crop‐cover and edge rasters using a lookup table converting this information to nesting quality, floral cover, and attractiveness values.

A season was divided into two floral periods, with the first period (late April–early June) corresponding to the flowering period of OSR, and the second (late June–late August) to the remaining part of the pollinator activity period. For each landscape, the raster of floral resources for period *k*,* F*_*k*_ was derived as the product between cell‐specific floral coverage, expressed as the proportion of area covered by flowering plants, and a score of the species‐specific attractiveness of the typical flowers in a land‐use category and season. We assume attractiveness scores to reflect both attractiveness per se and nutritional quality. Nesting resources, assumed constant over the season, were calculated as the product of land‐use specific nesting quality, NQ_land‐use_ (a score between 0 and 1), and the average number of bumble bee nests per cell for a cell of the best nesting quality, *n*_max_. Parameters for land use and species‐specific nesting quality and floral attractiveness were informed by expert judgment and literature resources, whereas values for floral coverage were either based on empirical estimates or expert judgment, depending on the availability of empirical data in the corresponding land‐use category. We refer to the Supporting information for details on region, landscape selection, GIS information sources, resource scoring, and the technical aspects on how resource scores for patches of land use and field edges were combined into single rasters.

### 2.2 Population dynamics and foraging of bumble bees

Through changes in the parameter values, our model can be applied to a range of life histories, including early‐ and late‐active solitary bees, and specifying parameters for multiple species in the input files allows modeling central‐place foraging pollinator communities. As applied here, our model is based on a simplified two‐stage seasonal model of common early‐active bumble bee species. Despite its simplicity, it can be used to represent important within‐season dynamics affected by seasonal patterns in flower availability. In spring, a fertilized bumble bee queen starts a colony at a suitable nesting site. The queen forages by herself to provision the nest in which she has laid eggs, until the first workers reach maturity. From then on, the queen stays in the nest and produces more workers before switching to producing new queens and males (Duchateau & Velthuis, 1988). In our model, these dynamics were captured by letting queens forage during the first floral period and a subset of workers during the second period. The number of workers in the second period is determined by the resources gathered by the queen in the first period, and the number of new queens produced at the end of the second period by the resources gathered by the foraging workers during that period. At the end of the flowering season, the new queens overwinter, and those that survive the winter disperse to new cells to nest.

For a given year, we denote by:

- **Q**, the matrix of the number of queens foraging in period *k* = 1, which is equal to the number of nests as there is one queen per nest,
- **W**, the matrix of the number of bumble bee workers in period *k* = 2,
- **Q**_E_, the matrix of the number of bumble bee queens at the end of the season.

#### 2.2.1 Foraging

The foraging model calculates the rate at which cell *i* is visited by foraging bees from cells *j* during period *k* as

(1) $${\text{VR}}_{j\to i}^{k}=X_{k,j}\frac{F_{i,k}e^{-d_{i,j}/\beta }}{\sum_{q=1}^{n}F_{q,k}e^{-d_{q,j}/\beta }}\rho_{F}^{d_{i,j}}$$

where $X={\left(X_{k,i}\right)}_{1\leq i\leq n}$ is the number of foraging bees nesting in cell *i*,* d*_*i,j*_ is the Euclidean distance between cells *i* and *j*, β is the mean dispersal distance for foraging, and ρ_F_ is the survival rate per meter during foraging. The denominator in Equation (1) weights the attractiveness of cell *i* compared to the total attractiveness of the cells in the landscape and by foraging distances. In this way, a cell further away from cell *j* compared to cell *i* but with higher floral resources compared to *i* can be receiving more visits (Fig. S2).

For computational reasons, the dispersal kernel is set to zero for a distance larger than the 99th quantile in the kernel in the nominator. The foraging model assumes that the bees have full information about the distribution of floral and nesting resources in this area.

#### 2.2.2 Resources collected when foraging

Under the simplifying assumption, shared with other pollinator models (Lonsdorf et al., 2009; Olsson et al., 2015), that there is no depletion of floral resources and therefore no competition for them, the resources collected per nest in nest of cell *i* during period *k* was calculated as

(2) $$R_{k,i}=\frac{X_{k,i}}{N_{i}}\sum_{j=1}^{n}F_{k,j}\frac{e^{-d_{i,j}/\beta }}{\sum e^{-d_{i,j}/\beta }}$$

with *N*_*i*_ the number of nests in cell *i*. The resources collected thus correspond to the distance‐weighted resource values from cells in which bees are nesting.

#### 2.2.3 Population growth during season

The growth for bumble bee workers in cell *i* in period *k* = 1 was calculated as:

(3) $$W_{i}=Q_{i}\cdot w_{\max}\cdot G\left(R_{1,i},a_{w},b_{w}\right)$$

where *w*_max_ is the maximal number of workers produced per queen, *G*(·, *a*, *b*) is the cumulative distribution function of a log‐normal distribution with median *a* and variance *b*. Thus, *a*_w_ corresponds to the resources needed for a queen to produce half of the maximum number of workers, and *b*_w_ accounts for the steepness of the curve, that is, the sensitivity of the growth to a change in the resources (Fig. S3). This function was chosen to capture a monotonic effect on aggregated resources from 0 up to an upper bound.

Similarly, the growth for new bumble bee queens in cell *i* in period *k* = 2 was given by

(4) $$Q_{E,i}=Q_{i}\cdot q_{\max}\cdot G\left(R_{2,i},a_{Q},b_{Q}\right)$$

where *q*_max_ is the maximal number of queens produced per colony, *R*_2,*i*_ is given by Equation (2), letting $X_{2,i}=p_{w}\cdot W_{i}$, where *p*_w_ is the proportion of foraging workers. Yet, to ensure the presence of pollinators in realistic landscapes while avoiding fully saturated landscapes before implementing the management interventions, *a*_q_ was set to a fixed value (Table 1; see Supporting information for details). For both growth functions, the variances *b*_w_ resp. *b*_q_ were fixed to be twice their corresponding median *a*_w_ resp. *a*_q_ (Table 1).

**Table 1:** Model parameters and parameter values used in the pollinator model

Parameter	Description	Unit	Value
*n*_max_	Number of nests in a cell of maximum nesting quality	nests/ha	19.6
β	Mean dispersal distance for foraging	m	530
$\tilde{\beta }$	Mean dispersal distance when flying to nesting sites	m	1,000
*a*_w_	Median of the growth rate for workers	—	100
*b*_w_	Steepness of the growth rate for workers	—	200
*a*_q_	Median of the growth rate for queens	—	15,000
*b*_q_	Steepness of the growth rate for queens	—	30,000
*w*_max_	Maximum number of workers that can be produced by a queen	—	600
*q*_max_	Maximum number of new queens produced	—	160
*p*_w_	Fraction of foraging workers	—	0.5

#### 2.2.4 Survival and dispersal between seasons

We assume that the new queens overwinter in the cell where they were produced, and then disperse from these cells *j* to new nesting cells *i* in early spring:

(5) $$Q_{E,j\to i}=Q_{E,j}\;\frac{Q_{i}e^{-d_{i,j}/\tilde{\beta }}}{\sum_{q=1}^{n}Q_{q}e^{-d_{k,j}/\tilde{\beta }}}\rho_{N}^{d_{i,j}}$$

where ρ_*N*_ is the survival rate per meter and $\tilde{\beta }$ is the mean dispersal distance to nesting sites, which we set to be around twice as large as the foraging distance (Table 1; Lepais et al., 2010).

We generate the number of new colonies $Q_{i}^{\prime }$ in cell *i* in two steps. First, we truncate $Q_{E,j\to i}$ at the maximum number of colonies per cell, which is the product of the NQ_land‐use_ and *n*_max_ (defined earlier), thereby introducing density‐dependent mortality among nest‐searching queens. Secondly, in order to get integer values for the number of cells, we draw the number of new colonies from a Poisson distribution with the intensity being the truncated number of queens obtained in the previous step.

#### 2.2.5 Pollination ecosystem services

For the purpose of illustration, pollination ecosystem services were simply quantified as the visitation rates per area of flowers per cell (floral coverage) for each floral period, and separately for OSR, which is early‐flowering. This simplifying assumption ignores pollination by other, nonmodeled species and nonlinear relationships between pollinator visitation and variables such as seed set and crop yield.

### 2.3 Management interventions

**Figure 2:**
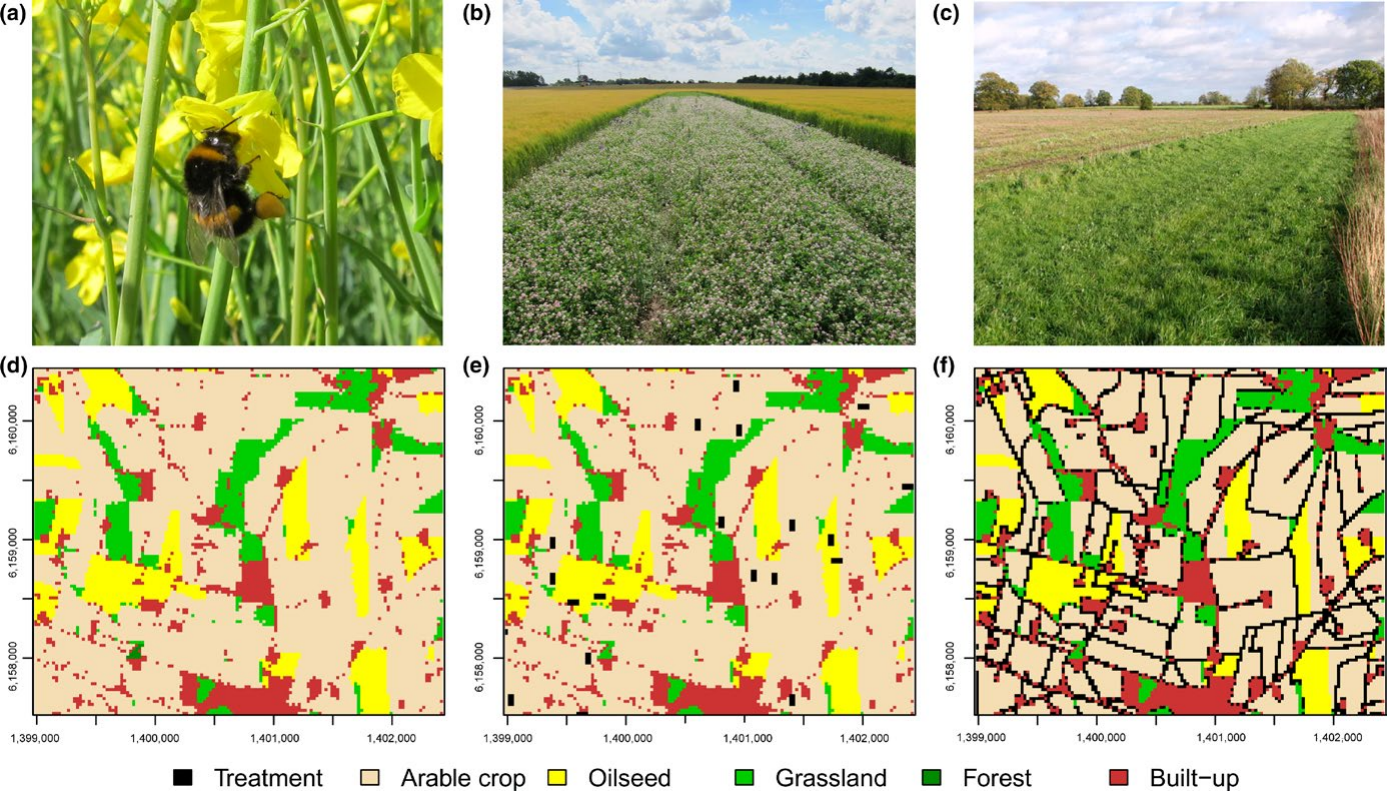
Study system and management interventions: Bumble bee *Bombus* sp. (a) on an oilseed rape flower, late‐flowering flower strip (b), wide grassy field margin (c), section of a landscape (d) with location of flowers strips (e), and wider field margins (f) highlighted in black. Landscape rasters are approximately 3 × 3 km in size. Photographs by Maj Rundlöf (a, b, with permission) and Evelyn Simiak (c, used under CCL)

Management interventions increasing availability of resources for pollinators were implemented across every raster. In total, we included four management alternatives in this study: (1) baseline (no intervention); (2) uncultivated, grassy field margins; (3) flower strips; and (4) grassy field margins and flower strips. To accommodate the area used for management crop field size is reduced which is accounted for in later calculations. To ensure comparability between the treatments for each landscape *l* and year *y*, the same cells were selected as flower strips in all relevant management interventions. We generated a close‐to‐optimal implementation across the landscape, using algorithms that place the resources where they would be most beneficial (see Supporting information for details). In this way, the resulting ecological production function predicts the outputs given the most optimal use of the inputs. The management interventions are illustrated in Figure 2, and were defined and implemented as follows.

#### 2.3.1 Field margins

Improve nesting quality by broadening the width of all uncultivated field block margins from 2.4 m (baseline scenario) to 4.8 m by adding an uncultivated permanent grass strip around fields. As field margins were characterized by maximum nesting quality and very low floral quality in both periods (Table S4), this increased the total nesting quality in the cells containing field block edges, while flower resources were either kept constant (e.g., in the case of cereal field borders) or decreased (in the case of mass‐flowering crop field borders).

#### 2.3.2 Flower strips

Increase floral resources in agricultural land by the creation of flower strips. A flower strip was implemented as a set of cells with high flower coverage late in the season and maximum bumble bee attractiveness (Table S4). The size of one flower strip was 25 × 50 m, and the number of flower strips per landscape was set to correspond to approximately 1.25% of the agricultural land, which correspond to a partial contribution toward the greening payment requirements of the new Common Agricultural Policy (EU regulation 1307/2013 Art. 46). On the assumption that agricultural land is plowed every year, flower strips are placed anew every year following an algorithm that is described in the Supporting information and thus have low nesting quality (Table S4).

### 2.4 Simulation and output

For each landscape, the foraging‐pollinator model was run for 35 years by repeating the 2007–2013 land‐use sequence five times. To level off bumble bee population sizes after initialization in the first year, the simulation was run without resource manipulations for each landscape over a cycle of seven land‐use maps as a burn‐in period.

For each year, landscape and management intervention the following response variables were computed for a 3‐km radius circular buffer around the center of the landscape: number of bumble bee colonies per ha (pollinator population size), average number of workers per colony (colony size), average number of new queens produced at the end of a season (reproductive success), visitation rates per unit flower cover per hectare for each early (pollination 1), and late‐flowering period (pollination 2), mean visitation rate per hectare in oilseed rape (OSR pollination). In addition, we computed the explanatory variables area of OSR and HET, defined as the length of field edges in the landscape.

### 2.5 Analysis

For each response variable, we fit a full linear mixed‐effect model with fixed effects and landscape and identity of the land‐use map within the 7‐year sequence as random effects. The latter is to avoid the sequence of the maps used to generate the 35‐year time series of land‐use bearing on the results. Fixed effects included dummy variables for management interventions, both landscape variables, year, as well as all possible interaction terms between management variables, between management variables and landscape variables, and between management variables and year. We present the models with the lowest AIC among all possible subset models fit using maximum likelihood. Model fits were checked for observations exerting extreme leverage (one instance, named in the results) and for variance heterogeneity (none detected). While we present *p*‐values in the results tables, we mainly discuss relative effect sizes, as the *p*‐values are driven by the arbitrary choice of the number of landscapes we use in the simulation. Thus, prior to analysis, all response variables as well as the explanatory variables OSR and HET, but not year, were *z*‐standardized to allow comparison of effect sizes between explanatory and response variables. As number of colonies in the first year are identical for all four treatment combinations, we excluded the first year for the analysis of number of colonies to avoid detecting spurious year × treatment interactions.

## 3 Results

Over all landscapes and years, the average area of increased field margins within a 3‐km radius (2,828 ha) around the center of the landscape was 32 ± 11 ha (mean ± *SD*; range 11–48 ha). The average area of agricultural land set aside as flower strips within the 3‐km radius was 19 ± 10 ha (mean ± *SD*; range 5–46 ha). Due to the differences in landscape composition, there is a high variation of area used for management between the landscapes we tested.

Landscape heterogeneity, measured as the area of edges in the baseline scenario within the 3‐km radius, ranged from 17 to 78 ha; the area of oilseed ranged from 14 to 323 (baseline), 316 (field margin), 217 (flower strip), or 209 ha (field margin plus flower strip scenario), respectively.

In the baseline scenario without management interventions, landscape heterogeneity and cover of oilseed rape had no effect on pollinator population size (Table 2). Very high heterogeneity values are partly found in areas dominated by agriculture with little flower resources in the second period, which is the most limiting resource. Cover of oilseed rape, which flowers in the first period when the colonies build up the number of workers but not in the second period when reproductives are produced, had a positive effect on colony growth, but not on reproductive success. Oilseed rape cover was associated with a much lower pollinator visitation rate per unit flower cover in the first period, and much higher visitation rates in the second period (Table 3). Due to dilution effects, visitation rate in oilseed rape was negatively affected by the cover of that crop in the landscape. The model for reproductive success shown in Table 2 was fit with one landscape left out (and the raw data re‐standardized after that). The extremely high density of new queens in that landscape exerted disproportionate leverage on the data and caused a negative effect of oilseed rape cover on reproductive success, for which there was no support in the rest of the data.

**Table 2:** Effect of management interventions, landscape composition, time, and their interactions on predicted pollinator population size and two measures of pollinator fitness

	Pollinator population size	Colony size	Reproductive success
Intercept	−.306 (.193)	−.213 (.125)	−.391 (.133)
	*t* = −1.585	*t* = −1.698	*t* = −2.939**
FM	.091 (.014)	.091 (.010)	.020 (.011)
	*t* = 6.327***	*t* = 9.301***	*t* = 1.803
FS	.404 (.031)	.230 (.020)	.690 (.018)
	*t* = 13.091***	*t* = 11.499***	*t* = 38.361***
HET	−.147 (.112)	−.047 (.085)	.192 (.113)
	*t* = −1.312	*t* = −.554	*t* = 1.700
OSR	.024 (.045)	.535 (.036)	.108 (.062)
	*t* = 0.526	*t* = 14.656***	*t* = 1.761
Year	−.0004 (.001)	−.0004 (.001)	−.00005 (.001)
	*t* = −0.334	*t* = −0.551	*t* = −0.085
FM × FS			.076 (.016)
			*t* = 4.786***
FM × HET		−.015 (.010)	−.011 (.011)
		*t* = −1.500	*t* = −0.943
FM × OSR	.072 (.014)	.099 (.010)	.025 (.011)
	*t* = 4.983***	*t* = 9.985***	*t* = 2.221*
FS × HET	.289 (.015)	.199 (.010)	.052 (.011)
	*t* = 19.705***	*t* = 19.968***	*t* = 4.549***
FS × OSR	−.196 (.015)	−.039 (.010)	.533 (.011)
	*t* = −13.404***	*t* = −3.927***	*t* = 46.923***
FS × year	.008 (.001)	.006 (.001)	.002 (.001)
	*t* = 5.582***	*t* = 6.690***	*t* = 2.744**
FM × FS × HET			−.027 (.016)
			*t* = −1.675
FM × FS × OSR			.095 (.016)
			*t* = 5.935***
Observations	2,720	2,800	2,660
*df*	2,571 (119)	2,649 (119)	2,516 (113)
AIC	2,821.261	846.493	−290.127

Results are from mixed‐effect models with identity of the land‐use map within the 7‐year sequence nested in landscape (site ID) as random effects. Given are coefficients, standard errors (in brackets), *t*‐values (*t*), and corresponding significance levels. Response variables, HET and OSR were *z*‐transformed prior to analysis. FM, field margins; FS, flower strips; HET, landscape heterogeneity; OSR, oilseed rape; *df*, degrees of freedom; AIC, Akaike information criterion. For heterogeneity, which does not change between years, degrees of freedom are given in brackets.

Significance levels: *p < .05; **p < .01; ***p < .001.

**Table 3:** Effect of management interventions, landscape composition, time, and their interactions on predicted visitation rates per area flower cover

	Pollination 1	Pollination 2	OSR pollination
Intercept	−.297 (.207)	.124 (.102)	−.349 (.193)
	*t* = −1.438	*t* = 1.224	*t* = −1.813
FM	.054 (.015)	.061 (.013)	.097 (.019)
	*t* = 3.564***	*t* = 4.612***	*t* = 5.187***
FS	.347 (.031)	−.408 (.027)	.381 (.038)
	*t* = 11.233***	*t* = −15.141***	*t* = 10.013***
HET	−.116 (.133)	−.036 (.089)	−.056 (.143)
	*t* = −0.870	*t* = −0.406	*t* = −0.393
OSR	−.267 (.056)	.742 (.051)	−.341 (.065)
	*t* = −4.799***	*t* = 14.593***	*t* = −5.216***
Year	.0005 (.001)	−.001 (.001)	.0001 (.001)
	*t* = 0.446	*t* = −1.363	*t* = 0.086
FM × OSR	.029 (.015)	.076 (.013)	.042 (.019)
	*t* = 1.901	*t* = 5.810***	*t* = 2.230*
FS × HET	.194 (.015)	.164 (.013)	.328 (.019)
	*t* = 12.650***	*t* = 12.290***	*t* = 17.356***
FS × OSR	−.228 (.015)	−.562 (.013)	−.314 (.019)
	*t* = −14.819***	*t* = −41.967***	*t* = −16.589***
FS × year	.010 (.001)	.008 (.001)	.012 (.002)
	*t* = 6.466***	*t* = 5.926***	*t* = 6.414***
Observations	2,800	2,800	2,800
*df*	2,653 (119)	2,652 (119)	2,653 (119)
AIC	3,268.946	2,512.081	4,424.583

Results are from mixed‐effect models with identity of the land‐use map within the 7‐year sequence nested in landscape (site ID) as random effects. Given are coefficients, standard errors (in brackets), *t*‐values (*t*), and corresponding significance levels. Response variables, HET and OSR were *z*‐transformed prior to analysis. FM, field margins; FS, flower strips; HET, landscape heterogeneity; OSR, oilseed rape; *df*, degrees of freedom; AIC, Akaike information criterion. For heterogeneity, which does not change between years, degrees of freedom are given in brackets.

Significance levels: *p < .05; **p < .01; ***p < .001.

**Figure 3:**
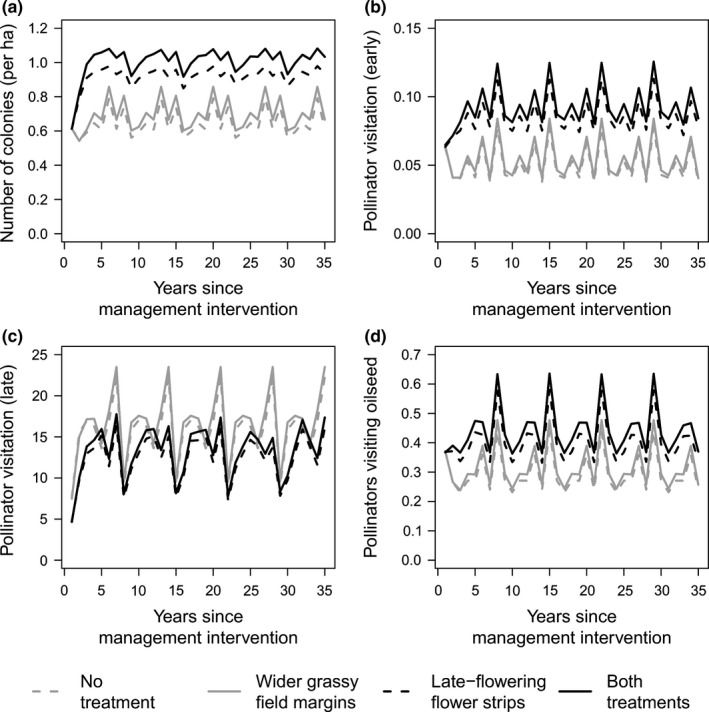
Effect of management interventions on the mean number of bumble bee colonies per hectare (a), pollinator visitation per flower cover per hectare early (b) and late (c) in the season, as well as on oilseed rape (d), depicted for a single landscape. Repetition in patterns across the 35 years time is due to the fivefold repetition of an underlying 7‐year time series which has slight year‐to‐year differences in resources due to crop rotation

**Figure 4:**
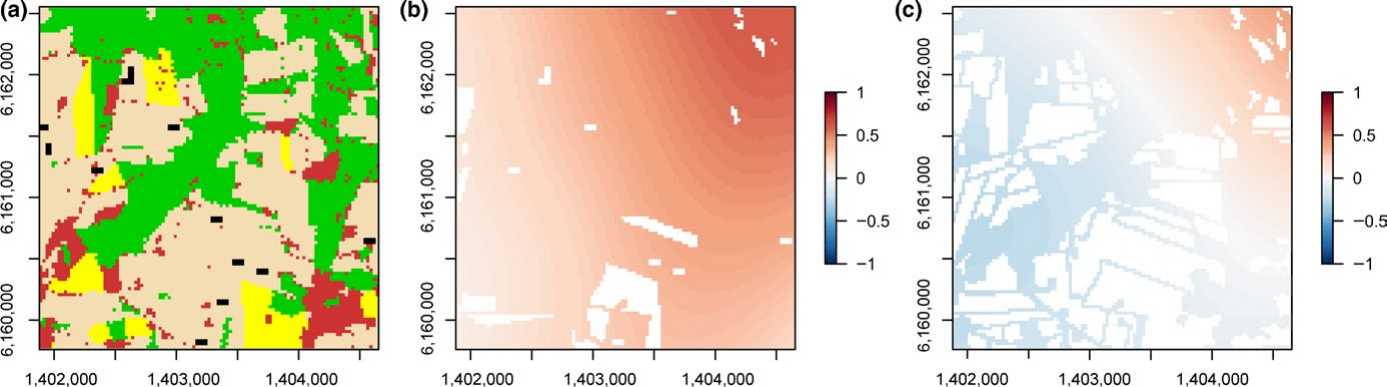
Effect size of flower strips (a) on early‐ and late‐season pollinator visitation (b, c), depicted as the log10‐response ratio

All response variables were stable across the time series in the baseline scenario. Establishment of wider grassy field margins and flower strips had significant positive effects on pollinator population size, colony growth, and reproductive success, as well as on early‐season pollination, including oilseed rape pollination (Table 2, Figures 3 and 4). The effect size of flower strips was a many‐fold larger than that of field margins (Table 2). Flowers strips had a strong negative effect on pollination in the second flowering period, however, when they attracted foragers away from existing flowering resources (Figure 4c). We further detected a time lag in the effect of flower strips on pollinator population size and pollinator fitness, with increased impact some years after implementing flower strips for the first time. The positive effect of flower strips on early‐season pollination increased over time, whereas the negative effect on late‐season pollination decreased over time. In contrast to the decreases observed after establishing flower strips, grassy field margins that offered increased nesting resources but no competing floral resources were associated with increases in late‐season pollination, an effect that increased over time.

Introducing field margins in addition to flower strips did not affect pollinator population size despite small positive effects on reproductive success, and had no significant effects on pollinator visitation, as this interaction was not included in the best models for these response variables (Table 3).

The effects of establishing flower strips and field margins were landscape‐dependent (Tables 2 and 3). In heterogeneous landscapes, the positive impact of flower strips on pollinator population size, colony size, reproductive success and early‐season pollination was substantially increased, while the negative effect of flower strips on late‐season pollination was strongly decreased, or even reversed. These effects are visible within‐landscape along the southwest–northeast gradient of increasing landscape heterogeneity and nonarable habitat in Figure 4. Wider field margins were more effective in increasing pollinator population size and early‐season pollination in landscapes rich in oilseed rape, but still less effective than flower strips (Table 3). The positive impact of wider field margins on increased late‐season pollination was larger in landscapes with a high cover of oilseed rape. The cover of oilseed rape in the landscape was associated with lower effects of flower strips, except for reproductive success, where the effect was reversed (Table 2).

## 4 Discussion

Pollinators respond to enhanced resources by redistributing from poorer to better resources, within the constraints set by their mobility. The resulting increase in accumulated resources led to a higher number of foragers later in the season, and in following years. The model we developed and applied here includes within and between‐year population dynamics and resource‐dependent foraging, and replicates patterns found in empirical studies. The application demonstrated that the model can give insight on how availability and spatial arrangement of resources affect bumble bees and the pollination ecosystem services they provide across spatial and temporal scales. Emergent patterns included in particular the partial offsetting of apparent competition for pollinators between established and already existing floral resources by landscape heterogeneity on the one hand, and the positive impact of increases in total floral resources on pollinator populations on the other.

In order to boost pollinator populations, pollinator fitness, and pollination ecosystem services in agricultural landscapes, it is important to identify which resource constitutes the limiting factor. Two findings show that bumble bees are limited by flower resources in our model: longer field edges in heterogeneous landscapes, and enhanced, wider grassy field margins had little effect on pollinators compared to flower strips, despite edges being assigned high nesting values. These results are consistent with empirical findings by Blake et al. (2011), where grassland buffer strips which lack wildflowers are less efficient in boosting pollinator populations compared to buffer strips with additional wildflowers as foraging resource. Furthermore, our results show little extra benefits are to be expected from a joint scheme combining flower strips and wider grassy field margins, over a scheme with only flower strips. In an approach to reverse the limiting effects of floral and nesting resources, we tested a reduced number of potential nesting sites (results not shown). Yet, even with a 50%‐reduced maximum number of nests, floral resources remained the limiting resource with close to maximum nesting saturation if additional floral resources are provided.

Bumble bee colonies need resources during their long period of activity, and late‐flowering flower resources have been suggested to be the most limiting (Rundlöf et al., 2014; Westphal, Steffan‐Dewenter, & Tscharntke, 2009) as this corresponds to the period when the resources are allocated to the production of reproductives, rather than workers. In our landscapes, oilseed rape is an important early mass‐flowering crop and flower resources are scarcer in the second half of the season after its flowering peak. Our findings are consistent with empirical studies which did not find a positive effect of increased cover of early mass‐flowering crops on bumble bee productivity despite increasing colony growth (Riedinger et al., 2015; Westphal et al., 2009; Williams, Regetz, & Kremen, 2012), and with studies showing significant benefits from the provision of additional floral resources in the second half of the season (Scheper et al., 2015).

Flower strips were very effective in increasing not only pollinator population size, but also, by extension, early‐season pollinator visitation, including to oilseed rape. In contrast, flower‐strip establishment resulted in lower pollination values for other floral resources flowering at the same time, that is, later in the season. This apparent competition between resources is visible in the reduced overall pollinator visitation rates in landscapes with higher oilseed rape cover, a pattern which has been demonstrated empirically using phytometers (Riedinger et al., 2014) and Europe‐wide field studies (Holzschuh et al., 2016). Interestingly though, consistent with empirical findings (Potts et al., 2009) we found a time lag in the emergence of an effect of management interventions on pollinator population size. This increase in pollinator population size over time in response to flower strips meant that the apparent competition effect difference was reduced with the number of years as flower strips had been first established. This effect occurred even though flower strips were modeled as being resown in different places every year.

While the effects of field margins on pollinator population size were less strong than those of flower strips, field margins were very beneficial in that they increased late‐season pollination. This was due to field margins increasing pollinator population size and colony sizes, but without competing for flower visitors with existing late‐season flower resources. This effect was increased further when oilseed rape cover was high, as this increased the size of colonies in field margins further.

More generally, our results support the landscape efficiency hypothesis stating that the effect of an intervention depends on landscape characteristics such as landscape heterogeneity (Persson, Olsson, Rundlöf, & Smith, 2010). The impact of increasing floral resources was more pronounced when additional nesting habitat was provided as well. Consequently, landscapes with a high structural heterogeneity fostered this effect further as heterogeneous landscapes often contain more seminatural habitats and more field edges, both associated with high nesting quality.

The conclusions with regard to which management option best supports bumble bees and flower visitation is not based on a cost‐benefit analysis, as effects on yield, and implementation and opportunity costs due to setting aside land for flower strips and grassy field margins have not been considered. Our model is a tool which produces key estimates to conduct such an analysis. We also emphasize that the findings of our model simulation study are dependent on the input parameters. While a sensitivity analysis is beyond the scope of the present paper, we consider the qualitative differences we observed as fairly robust. Having used expert opinion for the nesting and floral resource quality, however, means that the quantitative differences could change significantly as the parameter values are updated when more hard data becomes available.

The interaction of resource‐dependent foraging, apparent competition between flower resources and long‐term effects highlights the added value our model provides not only compared to the Lonsdorf model (Lonsdorf et al., 2009) but also over very recently developed models that approach optimal foraging (Olsson et al., 2015) and landscape‐scale resource‐dependent colony growth (Crone & Williams, 2016) without combining these two processes. Our model has several caveats, however, that could be alleviated by developing the model further. Most importantly, because we assumed that there is no depletion of floral resources, there is no competition for floral resources in our model, despite competition being common among pollinators (Pleasant, 1981). Competition between species could be taken into account by defining which species are most efficient, and running the model for each species sequentially, having the pollinator visitation of the most efficient species modify the resources available to the next‐most efficient species. Secondly, the effect of differential density of flowering plant species, and the individual movement choices of pollinators, addressed recently by Rands (2014) were not considered here. Thirdly, the current restriction to two flowering periods is limiting applicability to cases where higher resolution flower cover data are available or needed, but the structure of the model is kept fairly general and it can therefore be adapted for more periods. Finally, the model as applied in this study was parameterized for bumble bees. Results from Holzschuh et al. (2016) and Riedinger et al. (2015) suggest that solitary bees react quite differently to temporal changes in landscape composition including the availability of mass‐flowering crops. Hence, it remains to be seen how resource availability will affect other pollinator species and the pollination ecosystem services they provide. Our model is readily parameterized for other life histories, and the parameter files provided in the Supporting information contain preliminary parameter values for early‐active solitary bees (e.g., common early‐active *Andrena* spp.). In principle, managed pollinators such as honey bees could also be modeled with the present model by integrating apiary locations in the nesting quality input file and adapting the parameter values accordingly.

## Conflict of Interest

None declared.

## Data Accessibility

The code is available in the Supporting information, as are the data to run the model exemplarily on one of the 20 landscapes. The whole data will be archived on Dryad.
