# Supplementary material for: Pollinator population size and pollination ecosystem service responses to enhancing floral and nesting resources
Source: Ecol Evol. 2017 Feb 19;7(6):1898–908. doi: 10.1002/ece3.2765 (PMC5355185; doi:10.1002/ece3.2765)
Supplement: Supplementary file 1 [file ECE3-7-1898-s001.pdf]

Supplementary Information to the manuscript *"Predicting pollinator population size and pollination ecosystem service responses to enhancing floral and nesting resources"*

Johanna Häussler[a], Ullrika Sahlin[a], Charlotte Baey[a], Henrik Smith[a,b], and Yann Clough[a]

[a]Lund University, Centre for Environmental and Climate Research, SE-223 62 Lund, Sweden
[b]Lund University, Department of Biology, Biodiversity, Ecology Building, SE-223 62 Lund, Sweden

January 23, 2017

1 Region considered

Our model is parameterised for land uses and common crop-visiting bumble bees found in North, Northwest and parts of Central Europe. Conveniently, numerous empirical studies on bumble bees have been conducted in this region. The landscapes we run the model on are situated in southernmost Sweden, in the province of Skåne. An overview of the land-use in Scanian landscapes is given in Persson et al. (2010).

2 Pollinator species selection

Because the paper is focused on the provision of pollination services to early-flowering crops such as oilseed rape, we chose to consider a fictive bumble bee species that stands for several early-active species that are known to visit these crops in the region. These species are *Bombus terrestris*, *Bombus lucorum* and *Bombus lapidarius*. Besides being amongst the most important crop visiting bee species (Kleijn et al., 2015), they are also generally common in other habitats. For instance, these species made up two thirds of the number of bumble bees spotted in a large citizen survey conducted in the UK (Table 1 in Osborne et al. 2008). The selected species have several things in common: they have large colonies, large foraging ranges, and nest belowground (Hagen, 1994; Persson et al., 2015).

3 Choice of land-use classes

Our choice of land-use classes was driven by the data sources we used: the IACS (Integrated Administration and Control System) database which contains for each year the crops grown in each field block, and the Swedish Marktäcke Data (SMD), which is largely analogous to the CORINE Landcover data, including in the naming of the classes. This choice was motivated by

the fact that these or very similar data are available across Europe. More detailed habitat data would be very country or region-specific in attributes and classes.

The Swedish IACS is only partly spatially explicit in that it only contains the number of hectares of each crop per block and the total block area, but not the location of fields within field block. An algorithm was programmed to assign crops-within-block to cells-within-block. This results in contiguous fields within block. A random process is used to decide for each block whether fields are oriented along a North-South or a East-West axis.

We considered that suburban areas as identified in the SMD data are one third garden, and two thirds built-up (houses, roads etc.), which is an approximation based on satellite images assessed in Google Earth.

4 Choice of seasons

In the region, oilseed rape is the dominant mass-flowering crop, offering large amount of floral resources early in the season, around April-May. Arable-dominated regions contains much less flowers in the period between June and September, unless specific measures are taken, such as the establishment of wildflower strips. The cover of early vs. later flowering resources is of great interest for bumble bee population dynamics (Westphal et al., 2009; Riedinger et al., 2015) and therefore for the build-up of pollinator populations.

5 Scoring floral value and nesting quality

A preliminary expert assessment was conducted with Maj Rundlöf and Ola Olsson (Lund University) in late 2014 in which all the land-use classes were assigned scores for three variables: floral cover, floral attractiveness for bumble bees, and nesting quality. Floral cover and floral attractiveness furthermore were scored separately for the two periods (April-May and June-September). Land-use classes and corresponding floral and nesting values are listed in Table S4.

Floral cover was defined as the proportion area covered by flowers. Floral attractiveness was defined as a score ranging from zero (not at all attractive, never used) to 20 (very attractive, preferred over other flowers). Floral cover was multiplied by floral attractiveness to obtain the species-specific floral value scores. Nesting quality was defined as a score ranging from zero (totally unsuitable) to one (very suitable). Scoring was based on both personal experience of encountering nest-searching queens, as well as on the presence of suitable nesting substrate.

Information on the absolute and relative nesting densities in different habitats was retrieved from published results of a citizen survey of bumble bee nest densities conducted in the UK (Osborne et al., 2008), and transect survey of nest-searching queens from Sweden (Svensson et al., 2000). We found the correspondences between the land-uses in those papers and the classes used here. Plots of the expert estimates against literature findings were very good (Fig. S1). Unpublished work comparing floral cover data and expert assessments suggest that while very low floral cover values tend to be overestimated by experts, ranks are largely conserved. This suggests that for the illustrative purposes of this study the parameters are useful, but that further work is necessary to quantify floral value more soundly for further onward use.

6 Defining maximum nest densities

An overview of landscape-wide and habitat-specific nest densities reported in the literature is given in Table S1. Besides gardens (which we do not consider explicitly as a land-use class), the

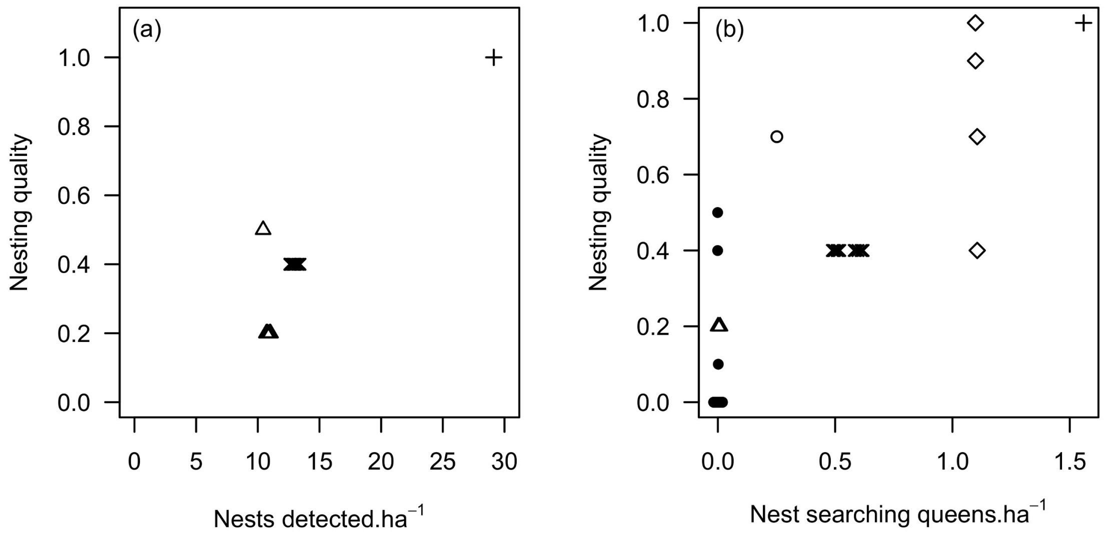

Figure S1: Nesting quality scores plotted against number of nests per ha detected in Osborne et al. (2008) and number of *Bombus terrestris*, *lapidarius* and *lucorum* queens detected searching for nests in Svensson et al. (2000). Filled circles are arable fields, standing crosses are edge habitats, crosses are grassland (pasture, leys), open triangles are woodland and forest habitats, open circles are forest clearings, open diamonds are other open habitat.

highest nest densities are observed in field edges, hedges and woodland edges (Table 2 in Osborne et al. (2008)). Since we do not distinguish between edge types in the current study, we used the mean density observed in the three edges as maximum nest density. We used the mean and not the maximum because we considered it likely that good bumble bee habitats were favored in the citizen survey. The maximum nest density was multiplied by the nest quality score (see above) to obtain the maximum nest density for the different land-classes. In addition, because the densities in Osborne et al. (2008) refer to total bumble bee nests rather than just those of the species we consider here, we multiply the maximum nest density for all bees by 0.66, because approximately two thirds of the nests found in the study could be assigned to the color groups corresponding to our species (Table 1 in Osborne et al. (2008)).

7 Foraging distance and shape of the dispersal kernel

We derived the choice of the dispersal kernel shape and the values for the mean dispersal distance from mark-recapture data for *B. terrestris* aggr. (includes *B. terrestris*, *B. lucorum* and *B. cryptorum*) and *B. lapidarius* published in Walther-Hellwig and Frankl (2000). We chose an exponential dispersal kernel and used a least-squares approach to estimate the mean dispersal distance from the quantiles given in the Figure of Walther-Hellwig and Frankl (2000), leading to $\beta_{foraging} = 530$. For illustration, a half of a one-dimensional kernel is shown in Figure S2.

Table S1: Landscape-wide and habitat-specific nest densities reported in the literature. Methods used were nest searches (Skovgaard, 1936; Osborne et al., 2008), and genetic markers (Darvill et al., 2004; Knight et al., 2005). Landscape-scale densities from Osborne et al. (2008) are upscaled from habitat-specific data.

Study	Habitat	Density (nests.ha^{-1})	Species	Region
Skovgaard (1936)	Different habitats	11-28	all	Denmark
Harder (1986)	Refuse dump	50	all	England
Darvill et al. (2004)	Landscape	0.13	*B. terrestris*	England
Knight et al. (2005)	Landscape	0.3	*B. terrestris*	England
Knight et al. (2005)	Landscape	1.2	*B. lapidarius*	England
Knight et al. (2005)	Landscape	2.4	Four common species	England
Osborne et al. (2008)	Landscape	7	all	England
Osborne et al. (2008)	Good nesting habitats	10-30	all	UK
Wood et al. (2015)	Landscape	0.1-1.7	*B. terrestris*	UK
Wood et al. (2015)	Landscape	0.1-0.7	*B. lapidarius*	UK

8 Population growth

The maximum number of workers ($w_{max} = 600$) produced is based on values for *B. terrestris* from Hagen (1994), which is consistent with the high-end values reported in other papers (Table S2). The maximum number of queens ($q_{max} = 160$) is based on Duchateau and Velthuis (1988). According to these authors, *B. terrestris* colonies are about equally distributed between two types, with early-switching colonies producing few workers, very large numbers of males and few or no queens, and late-switching colonies producing many workers, and both queens and males. We take the mean of the maxima (Duchateau and Velthuis, 1988) for these two types ($q_{max} = 160$). The growth functions for queens and workers as described in the main paper are illustrated in Figure S3.

As stated in the paper, we fixed the growth parameter a_q for bumble bee queens to

$$a_q = a_w p_w \frac{w_{max}}{2} \tag{1}$$

The value on a_q were chosen as a value small enough to ensure stable bumble bee populations in the landscapes included in our study and at the same time avoid landscapes that are fully saturated with bumble bees during the initialisation phase. In this way there were a pollinator population present in all study landscapes when the management interventions were implemented.

9 Foraging activity

We consider that 50 percent of the workers forage ($p_w = 0.5$) based on data listed in Table S3. Goulson et al. (2002b) reports that 31.6+-2.5 percent of *B. terrestris* workers from commercial nests were away from their nests. Free (1955) reports that only about a third of the workers forage

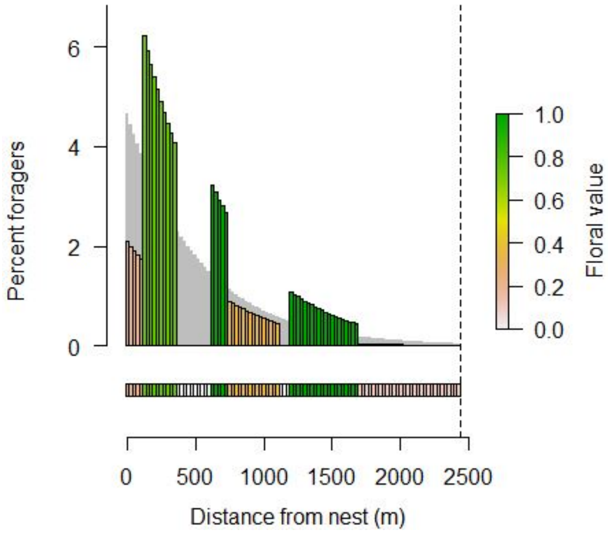

Figure S2: Proportion of foragers visiting a resource cell i at increasing distances $d_{i,j}$ from the nesting cell j, when floral values are equal (gray) or different (color-scale) across the landscape.

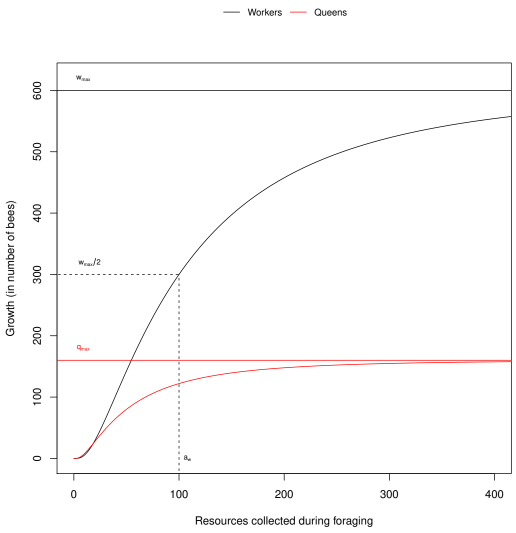

Figure S3: Growth functions giving the number of workers produced per queen during the first period (black curve) and the number of new queens produced at the end of the season (red curve), according to the proportion of workers produced compared to the maximum w_{max}.

Table S2: Worker numbers per nest reported in the literature.

Study	Habitat	Species	Estimate	Comment
Goulson et al. (2002a)	Gardens	*B. terrestris*	156.5 ± 20.0 (SE, n=10)	Commercial colonies
Goulson et al. (2002a)	Farm landscape, conventional	*B. terrestris*	167.0 ± 29.1 (SE, n=9)	Commercial colonies
Goulson et al. (2002a)	Farm landscape, diverse	*B. terrestris*	160.0 ± 23.4 (SE, n=9)	Commercial colonies
Duchateau and Velthuis (1988)	Unspecified	*B. terrestris* (early)	36.9 ± 58.8 (n=8)	Wild-caught queens
Duchateau and Velthuis (1988)	Unspecified	*B. terrestris* (late)	284.3 ± 145.0 (n=10)	Wild-caught queens
Hagen (1994)	Unspecified	*B. lapidarius*	100 to 320	Unspecified
Hagen (1994)	Unspecified	*B. terrestris*	100 to 600	Unspecified

more than 70 percent of the time, a third stay in the nest, and another third forages occasionally. Unfortunately relatively few nests were observed, only one very small one of *B. terrestris*, but the overall pattern was valid across species and nest sizes. Data from Brian (1952), consisting in daily observations of 2× 1h, conducted during 40 days for *Bombus pascuorum* (=*B. agrorum*) suggest that 30-55 percent of the workers foraged during the observation periods, with larger proportions observed only at the end of the season when drones had been produced and foragers had died off (55-72 percent foragers).

10 Dispersal of new queens, overwintering, and competition for nests

Little is known about the dispersal of new queens and the overwintering stage. Impacts of locally establishing flower resources have been detected at the same spot the year after (Scheper et al., 2015) suggesting that a significant part of the new queens nest in the vicinity of the old nest the following year. There is a large uncertainty in the distance the queens travel. Since we have estimates on the mean foraging distance, and the new queens may be expected to cover the same distance as a return trip to the a foraging we set $\beta_{nesting} = 1000$, i.e. about equal twice $\beta_{foraging}$.

There is evidence for competition for nesting sites among bumble bee species that use existing structures such as rodent holes to nest (McFrederick and LeBuhn, 2006). This applies to the species considered here. We therefore decided to cap the nesting densities at their maxima, considering that any additional queens will die.

Table S3: Percentage bumble bee workers foraging reported in the literature.

Study	% workers foraging	Colony development stage
Goulson et al. (2002b)	31.6%	Middle
Brian (1952)	30 to 55%	Early - middle
Brian (1952)	55-72%	late
Free (1955)	30-60%	Probably early

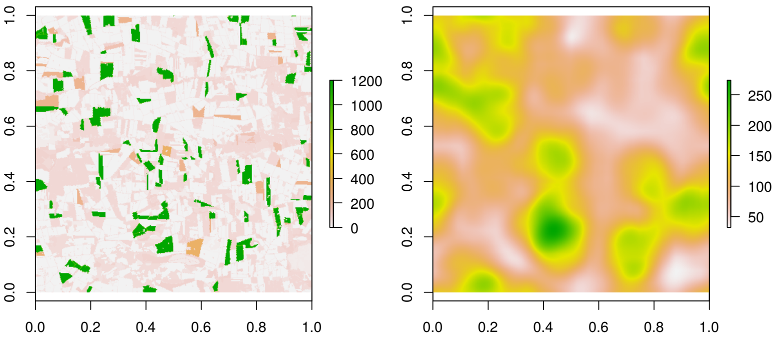

Figure S4: Weighting of floral resources (original values, left) in the landscape using a Gaussian kernel (weighted values, right). High values on the colour scale indicating a higher weighting.

11 Flower strip placement algorithm

The number of flower strips to be placed in the landscape is proportional to the percentage of agricultural land in the landscape, and their locations depend on the availability of resources nearby. More precisely, the probability for a flower strip to be placed at a given location is proportional to the ratio of floral resources in period 1 and period 2, with floral resources around a cell being computed using a Gaussian kernel (Fig. S4). Field edges and non-agricultural land are also excluded. Flower strips are placed sequentially in the landscape, and every time a new flower strip is settled, the probability for a cell to be selected as a future flower strip location is reduced according to the distance from the currently selected flower strip. An illustration of the algorithm is given in Figure S5.

See the R file `flowerStripsPlacement.R` for more details.

12 Landscape selection

To ensure uncorrelated gradients of landscape heterogeneity and proportion of oilseed rape in the landscape, we selected a subset of 20 10 $\times$ 10 km landscapes from an initial set of 43 landscapes. Using the D-optimality criterion (St. John and Draper, 1975), this subset of landscapes (Fig. S6) was chosen to be optimal to test linear, quadratic and interaction effects from temporal averages of oilseed rape area and landscape heterogeneity. By maximizing the determinant of the information matrix of the design, the D-optimality design creates the optimal set of experiments. It was implemented in the R-packaged AlgDesign (Wheeler, 2014) using the Federov algorithm.

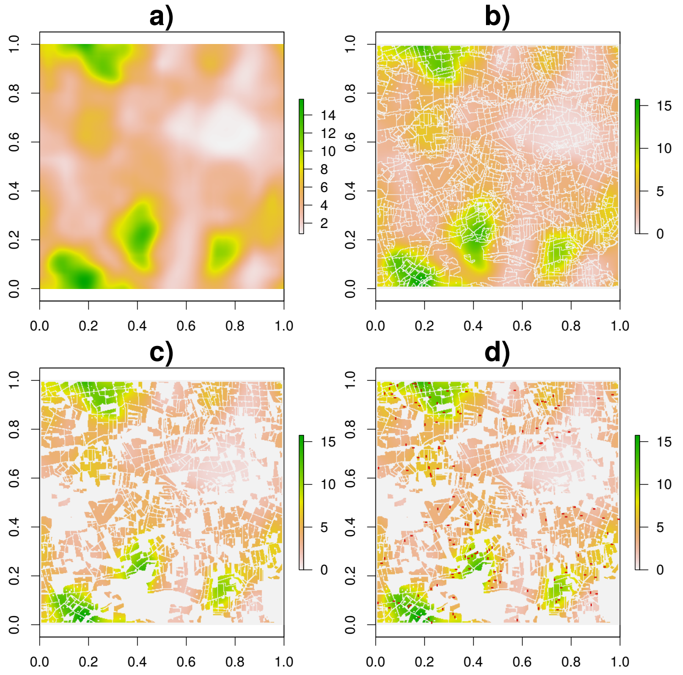

Figure S5: Flower strip placement algorithm: a) Weights are assigned to every cell of the landscape according to the ratio of floral resources in periods 1 and 2 (high values on the colour scale indicating a higher weighting), b) field edges are excluded from the set of possible locations, c) non-agricultural lands are excluded and d) final placement of flower strips in the landscape.

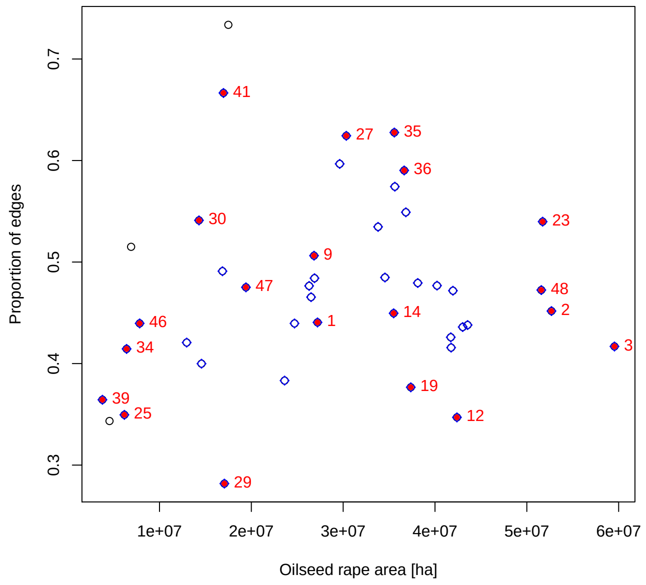

Figure S6: Relationship between the area of oilseed rape and landscape heterogeneity of the initial set of 43 landscapes. Landscapes with bumble bee population sizes above zero after leveling off population sizes after initialization are highlighted with blue borders. The subset of 20 landscapes included in our study which were selected using the D-optimality criterion are additionally highlighted in red and their corresponding landscape identification number.

Table S4: List of land-use classes and corresponding values of floral cover for each floral period, floral attractiveness for bumble bees for each floral period, and nesting attractiveness for bumble bees as well as the maximum number of nests per ha. Non-defined land-use classes are not included. Abbreviations: FC = Floral cover in period 1 or 2; FA = Floral attractiveness for bumble bees in period 1 or 2; NA = Nesting attractiveness for bumble bees.

Land-use code	Description	FC 1	FC 2	FA 1	FA 2	NA	Nests per ha
1	Barley (autumn)	0.01	0.01	5.00	0.00	0.00	0.00
2	Barley (spring)	0.02	0.01	5.00	0.00	0.00	0.00
3	Oats	0.02	0.01	5.00	0.00	0.00	0.00
4	Wheat (autumn)	0.01	0.01	5.00	0.00	0.00	0.00
5	Wheat (spring)	0.02	0.01	5.00	0.00	0.00	0.00
6	Mixed crops	0.02	0.01	5.00	0.00	0.00	0.00
7	Triticale	0.02	0.01	5.00	0.00	0.00	0.00
8	Rye	0.02	0.01	5.00	0.00	0.00	0.00
9	Maize	0.01	0.01	2.00	3.00	0.00	0.00
10	Buckwheat	0.10	80.00	5.00	10.00	0.00	0.00
11	Cereal trials	0.02	0.01	5.00	0.00	0.00	0.00
12	Mixed crops (grains)	0.02	0.01	5.00	0.00	0.00	0.00
13	Mixed crops (grains/legumes)	20.00	0.10	7.00	1.00	0.00	0.00
14	Canary grass	0.02	0.01	5.00	0.00	0.00	0.00
15	Millet	0.02	0.01	5.00	0.00	0.00	0.00
16	Grains for fodder	0.02	0.01	5.00	0.00	0.00	0.00
17	Bird field	0.02	0.01	5.00	0.00	0.00	0.00
18	Pasture[1]	0.19	0.11	12.00	12.00	0.40	0.00
19	Mown meadow[1]	0.19	0.11	12.00	12.00	0.40	0.00
20	Oilseed rape (autumn)	86.00	0.00	7.50	0.00	0.00	0.00
21	Oilseed rape (spring)	0.00	86.00	2.00	15.00	0.00	0.00
22	Turnip rape (autumn)	86.00	0.00	15.00	0.00	0.00	0.00
23	Turnip rape (spring)	0.00	86.00	2.00	15.00	0.00	0.00

Continued on next page

[1] no single payment scheme, no compensatory allowance

Table S4 – *Continued from previous page*

Land-use code	Description	FC 1	FC 2	FA 1	FA 2	NA	Nests per ha
24	Sunflower	0.10	80.00	5.00	18.00	0.00	0.00
25	Oilseed trials	86.00	0.00	15.00	0.00	0.00	0.00
26	High eruca rape	86.00	0.00	15.00	0.00	0.00	0.00
27	White mustard	0.02	0.01	0.00	0.00	0.00	0.00
28	Oil-seed (fodder) radish	0.00	86.00	2.00	15.00	0.00	0.00
30	Peas (non-tinned)	40.00	0.01	0.00	6.00	0.00	0.00
31	Peas (tinned)	40.00	0.01	0.00	6.00	0.00	0.00
32	Broad bean	0.10	50.00	5.00	15.00	0.00	0.00
33	Sweet lupin	0.10	50.00	5.00	15.00	0.00	0.00
34	Protein-rich mixed crops (legumes/grains)	20.00	0.10	7.00	1.00	0.00	0.00
35	Brown beans	0.10	50.00	5.00	15.00	0.00	0.00
36	Vetch	0.10	50.00	5.00	15.00	0.00	0.00
37	Chickpea	0.10	50.00	5.00	15.00	0.00	0.00
38	Soybean (oilseed)	0.10	50.00	5.00	15.00	0.00	0.00
39	Soybean (fodder)	0.10	50.00	5.00	15.00	0.00	0.00
40	Linseed	30.00	60.00	5.00	10.00	0.00	0.00
41	Flax	30.00	60.00	5.00	10.00	0.00	0.00
42	Hemp	0.01	0.01	5.00	0.00	0.00	0.00
44	Starch potato	40.00	40.00	0.00	6.00	0.00	0.00
45	Ware potato	40.00	40.00	6.00	6.00	0.00	0.00
46	Potato (processing)	40.00	40.00	0.00	6.00	0.00	0.00
47	Sugar beets	0.01	0.01	0.00	0.00	0.00	0.00
48	Fodder beets (mangel beets)	0.01	0.01	0.00	0.00	0.00	0.00
49	Non-approved ley	0.19	0.11	10.00	10.00	0.0	0.00
50	Ley	0.19	0.11	10.00	10.00	0.40	7.71
51	Ley[2]	0.19	0.11	10.00	10.00	0.40	0.00
52	Pasture	0.19	0.11	12.00	12.00	0.40	7.71
53	Mown meadow	0.19	0.11	12.00	12.00	0.40	0.00
54	Forest pasture	0.19	0.11	12.00	12.00	0.40	0.00

Continued on next page

[2] non eligible for agri-environmental scheme for cultivated grasslands

Table S4 – *Continued from previous page*

Land-use code	Description	FC 1	FC 2	FA 1	FA 2	NA	Nests per ha
55	Mountain pasture[3]	0.19	0.11	12.00	12.00	0.40	0.00
56	Alvar pasture	0.19	0.11	12.00	12.00	0.90	17.34
57	Ley (contracted with fodder drying)	0.19	0.11	10.00	10.00	0.40	0.00
58	Seed ley (annual)	17.00	17.00	20.00	20.00	0.10	1.93
59	Seed ley (perennial)	17.00	17.00	20.00	20.00	0.40	7.71
60	Fallow	7.00	7.00	10.00	10.00	0.40	7.71
61	Fallow (perennial)	0.19	0.11	12.00	12.00	0.40	0.00
62	Fallow (other)	7.00	7.00	10.00	10.00	0.40	0.00
63	Reed canary grass	0.01	0.01	2.00	2.00	0.40	7.71
64	Reed canary grass (other)	0.01	0.01	5.00	0.00	0.00	0.00
65	Salix	10.00	1.00	15.00	2.00	0.50	9.63
66	Adapted reduced leakage zone	0.19	0.11	10.00	10.00	0.40	0.00
67	Poplar	1.00	1.00	0.00	0.00	0.20	3.85
68	Hybrid aspen	1.00	1.00	0.00	0.00	0.20	0.00
69	Biodiversity fallow	8.00	8.00	12.00	12.00	0.40	7.71
70	Strawberry	3.00	2.00	15.00	0.00	0.20	3.85
71	Berries (other)	5.00	4.00	15.00	7.00	0.30	5.78
72	Fruits	20.00	1.00	15.00	7.00	0.30	0.00
73	Garden plants	10.00	10.00	4.00	4.00	0.20	3.85
74	Vegetable crops	0.01	0.01	0.00	0.00	0.00	0.00
75	Riparian strip	0.19	0.11	10.00	10.00	0.40	0.00
76	Riparian strip (other)	0.19	0.11	10.00	10.00	0.40	0.00
77	Riparian strip[4]	0.19	0.11	10.00	10.00	0.40	0.00
78	Nurseries for permanent crops	0.10	0.10	4.00	4.00	0.20	0.00
79	Aromatic herbs and vegetable seeds	10.00	10.00	10.00	15.00	0.20	3.85
80	Forage (green fodder)	0.19	0.11	10.00	10.00	0.40	0.00
81	Green manure	0.19	0.11	10.00	10.00	0.40	0.00
82	Wetlands	2.00	2.00	12.00	12.00	0.40	0.00

Continued on next page

[3] non-eligible for single payment scheme
[4] within agri-environmental scheme for riparian strips

Table S4 – *Continued from previous page*

Land-use code	Description	FC 1	FC 2	FA 1	FA 2	NA	Nests per ha
83	Christmas trees	1.00	1.00	0.00	0.00	0.20	0.00
84	Afforestation	1.00	1.00	10.00	10.00	0.80	15.41
85	Horticulture (non-household vegetables)	10.00	10.00	4.00	4.00	0.20	0.00
86	Non-eligible crop[5]	0.19	0.11	10.00	10.00	0.40	0.00
87	Other eligible crop[5]	0.19	0.11	10.00	10.00	0.40	0.00
88	Other landuse (arable field)	3.00	6.00	15.00	15.00	0.50	0.00
89	Other landuse (pasture)	0.19	0.11	12.00	12.00	0.40	0.00
91	Non-approved crop (arable field)	0.19	0.11	10.00	10.00	0.40	0.00
92	Non-approved crop (pasture)	0.19	0.11	12.00	12.00	0.40	0.00
93	Non-approved crop (other land)	0.19	0.11	10.00	10.00	0.40	0.00
94	Flooded land	0.19	0.11	12.00	12.00	0.40	0.00
95	Pasture under restoration[6]	0.19	0.11	12.00	12.00	0.40	0.00
96	Mosaic pastures[6]	0.19	0.11	12.00	12.00	0.40	0.00
97	Pasture[7]	0.19	0.11	12.00	12.00	0.40	0.00
98	Mown meadow[7]	0.19	0.11	12.00	12.00	0.40	0.00
99	Crop missing	0.19	0.11	10.00	10.00	0.40	0.00
100	Not Scania	0.00	0.00	0.00	0.00	0.00	0.00
101	City center	0.10	0.10	0.00	0.00	0.00	0.00
102	City urban	0.20	0.20	5.00	5.00	0.00	0.00
103	City suburb	2.00	2.00	10.00	10.00	0.40	7.71
104	Village	2.00	2.00	10.00	10.00	0.40	0.00
105	Rural settlement	2.00	2.00	10.00	10.00	0.40	0.00
106	Industrial area	0.10	0.10	0.00	0.00	0.00	0.00
107	Road railroad	3.00	3.00	10.00	10.00	0.40	0.00
108	Harbour	0.10	0.10	0.00	0.00	0.00	0.00
109	Airport	0.10	0.10	0.00	0.00	0.00	0.00
110	Sand gravel extraction	0.20	0.20	5.00	5.00	0.00	0.00

Continued on next page

[5] within agri-environmental scheme for organic farming
[6] within agri-environmental scheme for selected environment
[7] non-eligible for single payment scheme

Table S4 – *Continued from previous page*

Land-use code	Description	FC 1	FC 2	FA 1	FA 2	NA	Nests per ha
111	Mineral extraction	0.10	0.10	0.00	0.00	0.00	0.00
112	Dumps	0.20	0.20	5.00	5.00	0.00	0.00
113	Construction site	0.10	0.10	0.00	0.00	0.00	0.00
114	Green urban area	4.00	4.00	10.00	10.00	0.40	0.00
115	Sport leisure facility	0.10	0.10	0.00	0.00	0.00	0.00
117	Ski slope	0.19	0.11	12.00	12.00	0.40	0.00
118	Golf course	2.00	2.00	10.00	10.00	0.75	14.45
119	Non urban park	4.00	4.00	10.00	10.00	0.40	0.00
120	Camping holiday homes	2.00	2.00	5.00	5.00	0.00	0.00
130	Arable land	3.00	6.00	15.00	15.00	0.50	9.63
131	Permanent crop	0.01	0.01	5.00	0.00	0.00	0.00
132	Pasture	0.19	0.11	12.00	12.00	0.40	0.00
140	Deciduous woodland	5.00	0.50	12.00	0.00	0.20	3.85
141	Deciduous woodland mire	2.00	0.50	12.00	0.00	0.20	0.00
142	Deciduous woodland rock	5.00	0.50	12.00	0.00	0.20	0.00
143	Coniferous woodland lichen	2.00	0.10	10.00	0.00	0.20	3.85
144	Coniferous woodland (5-15)	0.00	0.00	0.00	0.00	0.00	0.00
145	Coniferous woodland (>15)	2.00	0.10	10.00	0.00	0.20	0.00
146	Coniferous woodland mire	2.00	0.10	10.00	0.00	0.20	0.00
147	Coniferous woodland rock	2.00	0.10	10.00	0.00	0.20	0.00
148	Mixed woodland	2.00	0.10	12.00	0.00	0.20	0.00
149	Mixed woodland mire	2.00	0.10	12.00	0.00	0.20	0.00
150	Mixed woodland rock	2.00	0.10	12.00	0.00	0.20	0.00
151	Natural grassland	0.19	0.11	12.00	12.00	0.40	0.00
152	Moorland	0.00	2.00	0.00	15.00	0.50	9.63
153	Scrub	1.00	3.00	5.00	15.00	0.70	13.49
154	Clearing	1.00	3.00	5.00	15.00	0.70	0.00
155	Young forest	0.00	0.00	0.00	0.00	0.00	0.00
156	Coniferous woodland	2.00	0.10	10.00	0.00	0.20	0.00
158	Beaches/dunes/sand planes	0.10	0.10	5.00	15.00	0.00	0.00

Continued on next page

Table S4 – *Continued from previous page*

Land-use code	Description	FC 1	FC 2	FA 1	FA 2	NA	Nests per ha
159	Bare rock	0.00	0.00	0.00	0.00	0.00	0.00
160	Sparsely vegetated area	0.10	0.10	5.00	15.00	1.00	19.27
161	Burnt area	0.00	0.00	0.00	0.00	0.00	0.00
162	Glaciers	0.00	0.00	0.00	0.00	0.00	0.00
163	Grassland	0.19	0.11	12.00	12.00	0.40	0.00
164	Meadow	0.19	0.11	12.00	12.00	0.40	0.00
170	Marshland	1.00	2.00	12.00	8.00	0.00	0.00
171	Wet mire	1.00	2.00	10.00	2.00	0.00	0.00
172	Mire	0.10	3.00	10.00	2.00	0.00	0.00
173	Peat extraction site	0.00	0.00	0.00	0.00	0.00	0.00
174	Salt marsh	0.10	0.10	2.00	2.00	0.00	0.00
180	Watercourse	0.00	0.00	0.00	0.00	0.00	0.00
181	Open lake/pond	0.00	0.00	0.00	0.00	0.00	0.00
182	Covered lake/pond	0.00	0.00	0.00	0.00	0.00	0.00
183	Coastal lagoon	0.00	0.00	0.00	0.00	0.00	0.00
184	Estuary	0.00	0.00	0.00	0.00	0.00	0.00
185	Open sea	0.00	0.00	0.00	0.00	0.00	0.00
186	Covered sea	0.00	0.00	0.00	0.00	0.00	0.00
200	Field margins	0.12	0.05	10.00	10.00	1.00	19.27
-	Flower strips	0.00	70.00	20.00	20.00	0.10	1.93

References

Brian AD. 1952. Division of labour and foraging in bombus agrorum fabricius. *The Journal of Animal Ecology* : 223–240.

Darvill B, Knight ME, Goulson D. 2004. Use of genetic markers to quantify bumblebee foraging range and nest density. *Oikos* **107**: 471–478.

Duchateau M, Velthuis H. 1988. Development and reproductive strategies in bombus terrestris colonies. *Behaviour* **107**: 186–207.

Free JB. 1955. The division of labour within bumblebee colonies. *Insectes sociaux* **2**: 195–212.

Goulson D, Hughes W, Derwent L, Stout J. 2002a. Colony growth of the bumblebee, bombus terrestris, in improved and conventional agricultural and suburban habitats. *Oecologia* **130**: 267–273.

Goulson D, Peat J, Stout JC, Tucker J, Darvill B, Derwent LC, Hughes WO. 2002b. Can alloethism in workers of the bumblebee, bombus terrestris, be explained in terms of foraging efficiency? *Animal Behaviour* **64**: 123–130.

Hagen Ev. 1994. Hummeln, bestimmen, ansiedeln, vermehren, schützen. *Naturbuchverlag Augsburg* .

Harder LD. 1986. Influences on the density and dispersion of bumble bee nests (hymenoptera: Apidae). *Ecography* **9**: 99–103.

Kleijn D, Winfree R, Bartomeus I, Carvalheiro LG, Henry M, Isaacs R, Klein AM, Kremen C, M'Gonigle LK, Rader R, et al. 2015. Delivery of crop pollination services is an insufficient argument for wild pollinator conservation. *Nature communications* **6**.

Knight ME, Martin AP, Bishop S, Osborne JL, Hale RJ, Sanderson RA, Goulson D. 2005. An interspecific comparison of foraging range and nest density of four bumblebee (bombus) species. *Molecular Ecology* **14**: 1811–1820.

McFrederick QS, LeBuhn G. 2006. Are urban parks refuges for bumble bees bombus spp.(hymenoptera: Apidae)? *Biological Conservation* **129**: 372–382.

Osborne JL, Martin AP, Carreck NL, Swain JL, Knight ME, Goulson D, Hale RJ, Sanderson RA. 2008. Bumblebee flight distances in relation to the forage landscape. *Journal of Animal Ecology* **77**: 406–415.

Persson AS, Olsson O, Rundlöf M, Smith HG. 2010. Land use intensity and landscape complexity—analysis of landscape characteristics in an agricultural region in southern sweden. *Agriculture, ecosystems & environment* **136**: 169–176.

Persson AS, Rundlöf M, Clough Y, Smith HG. 2015. Bumble bees show trait-dependent vulnerability to landscape simplification. *Biodiversity and Conservation* : 1–21.

Riedinger V, Mitesser O, Hovestadt T, Steffan-Dewenter I, Holzschuh A. 2015. Annual dynamics of wild bee densities: attractiveness and productivity effects of oilseed rape. *Ecology* **96**: 1351–1360.

Scheper J, Bommarco R, Holzschuh A, Potts SG, Riedinger V, Roberts SP, Rundlöf M, Smith HG, Steffan-Dewenter I, Wickens JB, et al. 2015. Local and landscape-level floral resources explain effects of wildflower strips on wild bees across four european countries. *Journal of Applied Ecology* **52**: 1165–1175.

Skovgaard O. 1936. Rødkloverens bestøvning. *Humlebier og Humleboer. Det Kongelige Danske Videnskabernes Selskabs Skrifter Naturvidenskabelig og Mathematisk Afdeling* **9**: 1–140.

St John RC, Draper NR. 1975. D-optimality for regression designs: A review. *Technometrics* **17**: 15–23.

Svensson B, Lagerlöf J, Svensson BG. 2000. Habitat preferences of nest-seeking bumble bees (hymenoptera: Apidae) in an agricultural landscape. *Agriculture, Ecosystems & Environment* **77**: 247–255.

Walther-Hellwig K, Frankl R. 2000. Foraging habitats and foraging distances of bumblebees, bombus spp.(hym., apidae), in an agricultural landscape. *Journal of Applied Entomology* **124**: 299–306.

Westphal C, Steffan-Dewenter I, Tscharntke T. 2009. Mass flowering oilseed rape improves early colony growth but not sexual reproduction of bumblebees. *Journal of Applied Ecology* **46**: 187–193.

Wheeler B. 2014. *AlgDesign: Algorithmic Experimental Design.* R package version 1.1-7.3. URL `https://CRAN.R-project.org/package=AlgDesign`

Wood TJ, Holland JM, Hughes WO, Goulson D. 2015. Targeted agri-environment schemes significantly improve the population size of common farmland bumblebee species. *Molecular ecology* **24**: 1668–1680.
